# Mid- and late-life lifestyle activities as main drivers of general and domain-specific cognitive reserve in individuals with Parkinson’s disease: cross-sectional and longitudinal evidence from the LANDSCAPE study

**DOI:** 10.1007/s00415-024-12484-0

**Published:** 2024-07-01

**Authors:** Anja Ophey, Kathrin Wirtz, Steffen Wolfsgruber, Monika Balzer-Geldsetzer, Daniela Berg, Rüdiger Hilker-Roggendorf, Jan Kassubek, Inga Liepelt-Scarfone, Sara Becker, Britt Mollenhauer, Kathrin Reetz, Oliver Riedel, Jörg B. Schulz, Alexander Storch, Claudia Trenkwalder, Karsten Witt, Hans-Ullrich Wittchen, Richard Dodel, Sandra Roeske, Elke Kalbe

**Affiliations:** 1 Department of Medical Psychology | Neuropsychology and Gender Studies, Center for Neuropsychological Diagnostic and Intervention (CeNDI), Faculty of Medicine and University Hospital Cologne, University of Cologne, Cologne, Germany; 2 German Center for Neurodegenerative Diseases (DZNE), Bonn, Germany; 3 Ethikkommission, Ludwig-Maximilians-Universität München, München, Germany; 4 Department of Neurology, University Medical Center Schleswig-Holstein, Christian Albrechts-University (CAU), Campus Kiel, Kiel, Germany; 5 Department of Neurology, Klinikum Vest, Recklinghausen, Germany; 6 Ruhr-University, Bochum, Germany; 7 Department of Neurology, University Hospital Ulm, Ulm, Germany; 8 German Center for Neurodegenerative Diseases (DZNE), Ulm, Germany; 9 Department of Neurodegenerative Diseases, Hertie Institute for Clinical Brain Research, Eberhard Karls University Tübingen, Tübingen, Germany; 10 IB-Hochschule, Stuttgart, Germany; 11 Department of Psychology, University of Calgary, Calgary, AB, Canada; 12 Paracelsus-Elena Klinik, Kassel, Germany; 13 Department of Neurosurgery, University Medical Center, Goettingen, Germany; 14 Department of Neurology, RWTH Aachen University Hospital, Aachen, Germany; 15 JARA Institute Molecular Neuroscience and Neuroimaging (INM-11), Juelich, Aachen, Germany; 16 Department Clinical Epidemiology, Leibniz Institute for Prevention Research and Epidemiology-BIPS, Bremen, Germany; 17 Department of Neurology, University of Rostock and German Center for Neurodegenerative Diseases (DZNE) Rostock/Greifswald, Rostock, Germany; 18 Department of Neurology, School of Medicine and Health Sciences and Research Center Neurosensory Science, University of Oldenburg, Oldenburg, Germany; 19 Department of Neurology, Evangelic Hospital Oldenburg, Oldenburg, Germany; 20 Department of Psychiatry and Psychotherapy, University Hospital Munich, Ludwig-Maximilians-University Munich, Munich, Germany; 21 Department of Geriatric Medicine, University Duisburg-Essen, Essen, Germany; 22 German Center for Neurodegenerative Diseases (DZNE), Tübingen, Germany

**Keywords:** Parkinson's disease, Lifestyle, Cognitive reserve, Cognitive dysfunction

## Abstract

**Background:**

Cognitive reserve (CR) is considered a protective factor for cognitive function and may explain interindividual differences of cognitive performance given similar levels of neurodegeneration, e.g., in Alzheimer´s disease. Recent evidence suggests that CR is also relevant in Parkinson’s disease (PD).

**Objective:**

We aimed to explore the role of life-stage specific CR for overall cognition and specific cognitive domains cross-sectionally and longitudinally in PD.

**Methods:**

The cross-sectional analysis with data from the DEMPARK/LANDSCAPE study included 81 individuals without cognitive impairment (PD-N) and 87 individuals with mild cognitive impairment (PD-MCI). Longitudinal data covered 4 years with over 500 observations. CR was operationalized with the Lifetime of Experiences Questionnaire (LEQ), capturing the complexity of lifestyle activities across distinct life-stages. Cognition was assessed using a comprehensive neuropsychological test battery.

**Results:**

Higher LEQ scores, particularly from mid- and late-life, were observed in PD-N compared to PD-MCI [*F*(1,153) = 4.609, *p* = .033, *η*_p_^2^ = 0.029]. They were significantly associated with better cognitive performance (0.200 ≤ *β* ≤ 0.292). Longitudinally, linear mixed effect models (0.236 ≤ marginal *R*^2^ ≤ 0.441) revealed that LEQ scores were positively related to cognitive performance independent of time. However, the decline in overall cognition and memory over time was slightly more pronounced with higher LEQ scores.

**Conclusions:**

This study emphasizes the association between complex lifestyle activities and cognition in PD. Data indicate that while CR might be related to a delay of cognitive decline, individuals with high CR may experience a more pronounced drop in overall cognition and memory. Future studies will have to replicate these findings, particularly regarding domain-specific effects and considering reverse causal mechanisms.

**Supplementary Information:**

The online version contains supplementary material available at 10.1007/s00415-024-12484-0.

## Introduction

Cognitive decline is a common and highly debilitating non-motor symptom in Parkinson’s disease [PD; 1]. The prevalence of mild cognitive impairment (MCI) in PD (PD-MCI) is estimated around 40% [2]. Compared to those with PD without cognitive impairment (PD-N), individuals with PD-MCI have longer disease durations, higher levodopa equivalent daily doses (LEDD), and experience more severe motor and non-motor symptoms [2]. Moreover, individuals with PD-MCI have an increased risk of progressing to PD-dementia [PDD; 3]. Nevertheless, the trajectories of cognitive functioning in individuals with PD are highly heterogeneous [1, 3]. Understanding the factors that modulate cognitive performance and cognitive trajectories in PD and contribute to this heterogeneity can have important implications for prevention and intervention strategies. One such factor of interest is cognitive reserve (CR).

CR is a theoretical concept referring to the brain's ability to optimize or adapt cognitive processes to cope with brain pathology [4, 5]. It helps to explain interindividual differences in cognitive functioning given similar levels of brain pathology: The higher the CR, the better one’s cognitive performance [4, 5]. Unlike brain reserve, CR is an active concept, influenced by lifetime exposure to complex lifestyle activities [5]. The concept of CR was initially established in the context auf Alzheimer’s disease [AD; [6, 7]], however, it is increasingly recognized in PD as well. In a recent review and meta-analysis of studies on the association of CR and cognitive functioning in PD, higher educational levels were associated with better cognitive functioning and a lower risk of longitudinal progression of PD-N to PD-MCI. However, these studies also point out that many open questions remain [8]: For example, the relation between cognitive functioning and CR indicators is explored with educational levels or years of education as CR proxies in the majority of studies. Moreover, most longitudinal studies do not provide information on the trajectories of and timing information on cognitive decline.

Years of education have frequently been employed as a proxy measure to quantify CR [8, 9]. However, the unidimensionality of this proxy has faced criticism due to the multidimensional nature of the CR construct itself. Hence, multidimensional questionnaires have been developed, which consider factors beyond educational experiences, such as occupational complexity, engagement in diverse lifestyle activities, and social involvement [9, 10]. These measures consistently demonstrate a positive association between CR and cognitive performance [11–13]. One critique of multidimensional measures of CR is that they blur the conclusion regarding whether they function as a causal factor for cognitive preservation or simply reflect reverse causation: If complex lifestyle activities serve as a proxy for CR, we have to consider that people may naturally reduce those activities not only when they are already experiencing cognitive decline but potentially in the prodromal phase of cognitive decline and any condition associated with this decline as well [4]. Furthermore, not only are multidimensional measures of CR susceptible to reverse causation or “the chicken-egg” problem, so is education when used as a proxy for CR, as recently discussed by Kremen, et al. [14]. Measures of general cognitive ability (i.e., intelligence) administered during young adulthood have been shown to significantly diminish the prognostic value of education, occupational complexity and complex lifestyle activities later in life.

Beyond protective effects on global cognition, CR appears to have the greatest impact on cognitive domains that are particularly susceptible to decline in PD [11, 12, 15], e.g., executive functions [16, 17]. However, no protective effect of a more active lifelong cognitive lifestyle compared with a less active one on performance in tests of executive functions for individuals with PD was found in a cross-sectional study using the Lifetime of Experiences Questionnaire [LEQ; [18]] as a proxy for CR [19]. Larger longitudinal studies are needed to explore the potentially differential effects of CR in different cognitive domains in PD.

On the basis of the aforementioned considerations, the aim of this study is to evaluate the association of a well-established, multidimensional measure of CR, the LEQ, with the presence of cognitive impairment and general cognitive functioning across multiple cognitive domains in individuals with PD both cross-sectionally and longitudinally. The application of the LEQ as a multidimensional measure of CR (well aware of its limitations regarding causal interferences) will foster a more detailed understanding of which specific life-stages and lifestyle activities contribute to the potentially protective effect of CR on cognition in PD, which has not been investigated so far. Most longitudinal studies of CR and cognitive trajectories in PD relied on unidimensional approaches to operationalize CR [8] and have a limited number and time-frame of follow-ups [20]. Furthermore, the analysis of multiple cognitive domains both cross-sectionally and longitudinally will contribute to our understanding to the potentially heterogeneous effects of CR on different cognitive domains [8, 19] given the heterogeneous cognitive profiles of individuals with PD per se [1, 17]. Previous studies frequently focused on the progression from PD-N to PD-MCI or PDD [8], neglecting the dynamic nature of the PD-MCI diagnosis as some individuals transition back to PD-N [3]. The use of continuous measures of cognitive functioning — globally and domain-wise — may capture the full spectrum of cognitive changes [8]. For these purposes, we utilized data from the German DEMPARK/LANDSCAPE project, a multicenter, prospective, observational cohort study, which was designed to investigate the natural course of cognitive decline in individuals with PD [21].

## Methods

### Dempark/landscape study and participants

For the German DEMPARK/LANDSCAPE project [21], a total of 711 individuals with PD were consecutively recruited from nine movement disorder centers across Germany. These individuals underwent comprehensive clinical and cognitive assessments annually over a period of six years. The study's inclusion criteria required participants to be 45–80 years old at the initial assessment and have a diagnosis of idiopathic PD based on the UK Parkinson's Disease Society Brain Bank criteria [22].

For the present analyses, we selected those individuals of the DEMPARK/LANDSCAPE study with available LEQ data and excluded those with a diagnosis of PDD at the time of LEQ assessment. PDD [23] was defined by the following criteria: (i) criteria for PD according to the Queen Square Brain Bank, (ii) gradual onset and slow progression of cognitive deficits, (iii) impairment in at least two cognitive domains, with one test per domain being ≤ 1.5 SD below the mean of published normative data, (iv) impairment represented a decline from a premorbid level, and (v) impact of the experienced cognitive deficits on daily life independently of motor or autonomic symptoms as assessed by medical history. The LEQ was incorporated into the study protocol after the initiation of the DEMPARK/LANDSCAPE study and was administered either at the 2-year or 3-year follow-up. A total of 169 individuals, constituting 25.7% of the initial DEMPARK/LANDSCAPE cohort, completed the LEQ at the 2-year or 3-year follow-up and did not fulfill the diagnostic criteria for PDD at LEQ baseline, forming the sample for our cross-sectional analyses.

**Fig. 1:**
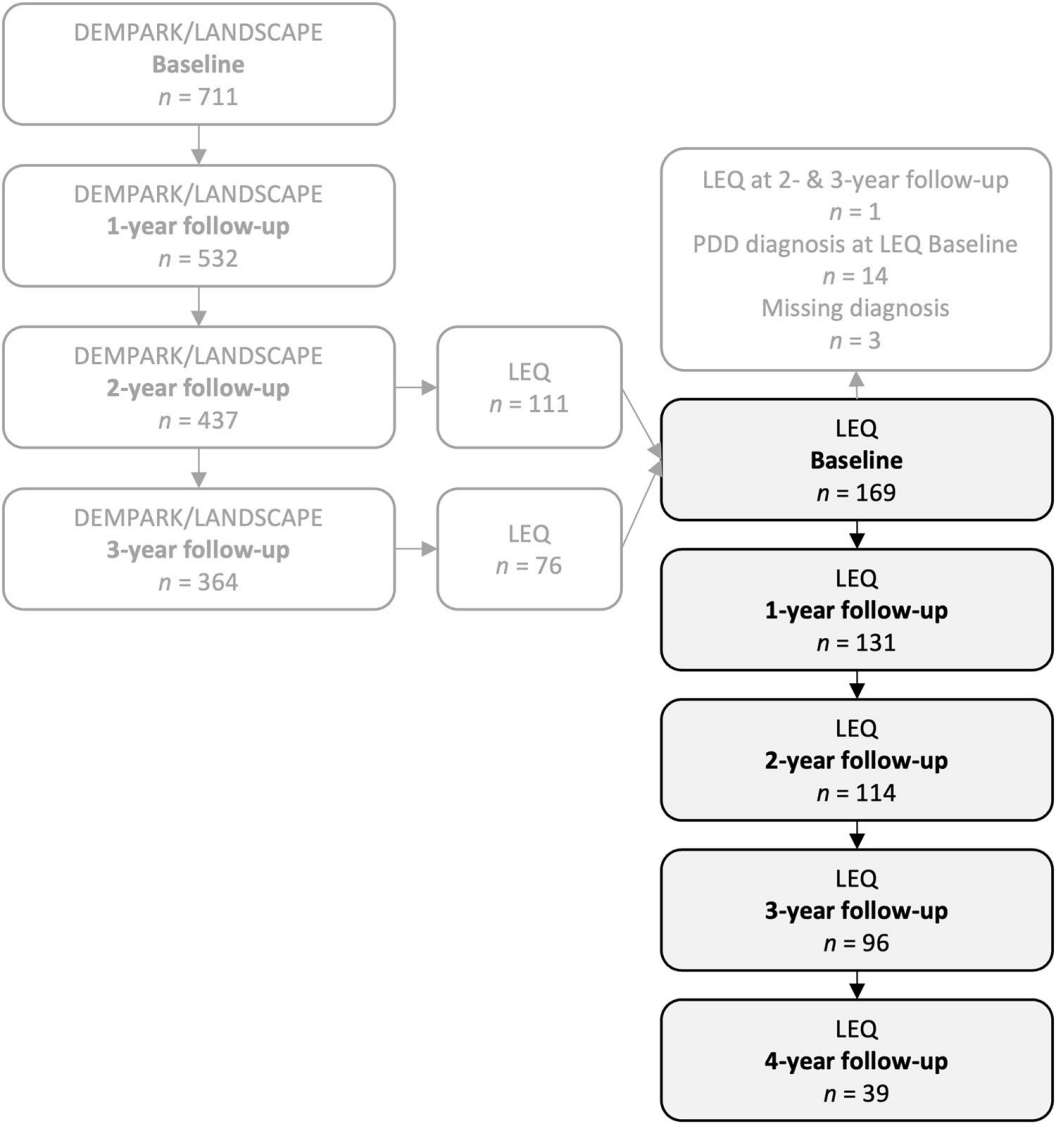
Flow of participants for the present analyses. Flow of participants in the DEMPARK/LANDSCAPE study and data selection for the present analyses based on individuals with available Lifetime of Experiences Questionnaire (LEQ) data and exclusion of Parkinson’s disease dementia (PDD) diagnosis

For the longitudinal analyses, the (first) time of the individual LEQ assessment was designated as baseline and the annual re-assessments following this “LEQ baseline” as corresponding follow-ups. This resulted in a maximum of four years of follow-up for those who completed the LEQ at the 2-year follow-up of the DEMPARK/LANDSCAPE study and three years of follow-up for those who completed the LEQ at the 3-year follow-up. The flow of participants throughout the time points is presented in Fig. 1.

### Clinical and neuropsychological assessment

Clinical assessment included the documentation of the disease duration defined as the time since initial PD diagnosis, the age at PD diagnosis, the LEDD [24], and the assessment of the severity of motor impairment, using the Unified Parkinson’s Disease Rating Scale Part III [UPDRS-III; 25]. Depressive symptoms were assessed with the Geriatric Depression Scale [26]. All assessments were performed during *ON* medication state.

All individuals underwent cognitive screening using the Mini Mental State Examination [27] and the Parkinson Neuropsychometric Dementia Assessment [28]. Furthermore, the comprehensive neuropsychological test battery included assessments of executive functions (semantic and phonemic verbal fluency, Modified Card Sorting Test, digit span backward), memory (word list learning and recall, figure recall, digit span forward), attention (Stroop Test, Brief Test of Attention), visuospatial functioning (figure copy, spatial rotation, spatial imagination), and language (Boston Naming Test). Details of the neuropsychological test battery can be found in Supplementary Table S1. For all five domains, cognitive domain composite scores were created by calculating the equally weighted mean of individual *z*-scores within each domain [16]. Previously, raw scores were *z*-standardized using the mean and SD of the PD-N sample at LEQ baseline (note, that these are not the *z*-scores on which the cognitive diagnosis is based [16]). The cognitive domain composite scores were not demographically adjusted. As a measure for overall cognitive function, the CERAD-Plus total score [29] was computed as an equally weighted mean score of the demographically adjusted *z*-scores of all subtests included in the German CERAD-Plus [30].

### Classification of cognitive status

The individuals’ cognitive status at LEQ baseline was defined following diagnostic criteria for cognitive impairment in PD available at time of the initial set-up of the DEMPARK/LANDSCAPE study [31] resulting in the categories of “no cognitive impairment” (PD-N) and PD-MCI. Individuals were classified as PD-MCI [31], if they met the following criteria: (i) self-reported subjective cognitive dysfunctions, (ii) no significant impairment in daily life activities, (iii) performance on at least one cognitive test relevant for the diagnosis of PD-MCI (for details, see Supplementary Table S1) was ≤ 1.5 SD below the mean of published normative data, (iv) exclusion of PDD according to the criteria described above [23].

### Assessment of cognitive reserve: the lifetime of experiences questionnaire

The LEQ [18], a well-established instrument assessing the complexity of educational, occupational and cognitive lifestyle activities across the lifespan, was used to assess CR. The original Australian version of the LEQ [18] was translated to German and was adapted to align with the German school system and occupational histories. The LEQ has been shown to be a stable and valid instrument across countries [32]. It comprises 42 questions, which encompass a combination of multiple-choice questions, 5-point Likert scale questions and free responses. The LEQ evaluates both specific and non-specific mental activities across three life-stages: young adulthood (13–30 years), mid-life (30–65 years), and late-life (from 65 years or retirement).

Life-stage specific questions in the young adulthood section primarily pertain to educational experiences (school and occupational training). In the mid-life section, they focus on the individual’s occupational history, with each occupation classified into one of the ten categories according to the Australian Standard Classification of Occupations [18]. In the late-life section, the questions revolve around current social and intellectual activities (e.g., living situation, volunteering, memberships, daily activities, media usage, continuing education). The non-specific questions for the three life-stages encompass questions about the participation and frequency of various activities such as travel, visiting family, playing musical instruments, doing arts, physical activities, reading, and speaking a foreign language [18].

By equally weighting specific and non-specific scores from each of the three life-stages (for details, see Supplementary Material), three subscores are constructed: young adulthood, mid-life, and late-life. Each subscore combines both the specific and non-specific scores, with each subscore contributing 33.3% to the overall LEQ total score. The higher the LEQ subscores and the LEQ total score, the higher the estimated CR [18].

### Statistical analysis

Statistical analyses and data visualization were conducted in R [33].

#### Cross-sectional analyses

For the baseline comparison of demographic, clinical, and neuropsychological data between the groups of PD-N and PD-MCI as well as individuals lost to attrition and those who completed their last possible follow-up, independent sample *t*-tests or *χ*^2^-tests were performed. To assess differences in CR based on cognitive status, ANCOVAs were conducted with cognitive status (PD-N vs. PD-MCI) as the between-subjects factor, and age, sex, UPDRS-III, and disease duration as covariates. The LEQ total score and three subscores were used as dependent variables. Multiple linear regressions were performed to examine the independent association of CR derived from the three LEQ life-stages on cognitive performance in global cognition and the five cognitive domains. Age, sex, UPDRS-III, disease duration, and depressive symptoms were included as covariates. LEQ scores and age were mean-centered prior to model estimation.

#### Longitudinal analyses

To analyze the association between CR and cognitive trajectories over time, repeated-measures linear mixed-effects (LME) models were estimated using the *lmer()* function of the *lme4*-package employing restricted maximum likelihood estimation [34]. Dependent variables were either the CERAD-Plus total score or the cognitive domain composite scores, each assessed at a maximum of five time points. The LME models included time since baseline (0–4 years), the LEQ total score, and the interaction between time and LEQ total score (time*LEQ) as fixed factors. Furthermore, the models were adjusted for age at LEQ baseline (in years), sex (male = 0, female = 1), the cognitive diagnosis at LEQ baseline (PD-N = 0, PD-MCI = 1), disease duration at LEQ baseline (in months), the UPDRS-III score at LEQ baseline, and depressive symptoms at LEQ baseline. LEQ scores and age were mean-centered prior to model estimation. Subjects and time were included as random factors in the models. A sensitivity analysis was conducted, including observations up to 3 years from the LEQ baseline only.

Model fit was evaluated using marginal *R*^2^, which considers the variance of the fixed effects only, and conditional *R*^2^, which accounts for both fixed and random effects. *t*-tests were conducted to assess the significance of single coefficients. No imputation methods were used, as one strength of LME models is their ability to deal with unbalanced designs, for example due to missing values in longitudinal data.

## Results

### Sample characteristics

Our final sample consisted of *n* = 169 individuals with PD, of which *n* = 81 were classified as PD-N, and *n* = 88 as PD-MCI. On average, individuals were 70.97 ± 6.45 years old, and 71.0% were male. Individuals with PD-N were significantly younger than individuals with PD-MCI, *t*(167) = − 2.0, *p* = 0.047. Total years of formal education did not significantly differ between the groups, *t*(167) = 0.38, *p* = 0.701. Individuals with PD-MCI had more severe motor impairment [*t*(159) = -2.53, *p* = 0.012], and more severe depressive symptoms [*t*(164) = − 2.58, *p* = 0.011] compared to PD-N. Across all cognitive tests, individuals with PD-N performed significantly better than individuals with PD-MCI [*ps* ≤ 0.002]. Further details are displayed in Table 1. Details on the composition of the LEQ (sub-)scores (life-stage x specificity) are reported in the Supplementary Material including Supplementary Figure S1.

**Table 1:** Sociodemographic, clinical, and cognitive characteristics of the DEMPARK/LANDSCAPE sample at LEQ baseline

	ALL (*n* = 169)	PD-N (*n* = 81)	PD-MCI (*n* = 88)	*p*-value
Age *in years*	70.67 (6.5) [50–85]	69.64 (7.1) [50–82]	71.62 (5.77) [56–85]	.047
Sex, *n (%)*	M: 120 (71.0) F: 49 (29.0)	M: 52 (64.2) F: 29 (35.8)	M: 68 (77.3) F: 20 (22.8)	.089
Education *in years*	14.24 (2.93) [8–20]	14.33 (2.99) [8–20]	14.16 (2.89) [8–20]	.701
PANDA	24.06 (4.55) [8–30]	25.74 (3.78) [13–30]	22.52 (4.67) [8–30]	< .001
MMSE	28.64 (1.39) [22–30]	29.01 (1.06) [26–30]	28.28 (1.57) [22–30]	< .001
Disease Duration *in months*	98.63 (48.27) [25–308]	95.16 (38.96) [33–199]	101.86 (55.59) [25–308]	.376
Age at PD Diagnosis *in years*	62.38 (7.47) [42–75]	61.72 (7.66) [42–74]	63 (7.28) [44–75]	.267
UPDRS-III	24.78 (11.91) [3–60]	22.34 (10.73) [3–56]	27.01 (12.54) [3–60]	.012
LEDD	655.58 (384.09) [52–2120]	655.23 (379.76) [52–1810.25]	655.9 (390.21) [80–2120]	.991
Depressive Symptoms (GDS)	3.01 (2.88) [0–15]	2.42 (2.17) [0–11]	3.56 (3.33) [0–15]	.011
CERAD-Plus Total Score	0.03 (0.63) [−2.41 – 1.44]	0.37 (0.45) [−0.78–1.44]	−0.29 (0.61) [−2.41–1.08]	< .001
Executive Functions composite	−0.63 (0.79) [−2.56–1.13]	−0.11 (0.58) [−1.41–1.13]	−1.11 (0.66) [−2.56–0.75]	< .001
Memory composite	−0.4 (0.82) [−3.09 –1.27]	−0.01 (0.68) [−1.8–1.27]	−0.77 (0.78) [−3.09–0.65]	< .001
Attention composite	−0.55 (1.16) [−4 –1.33]	−0.07 (0.8) [−2.97–1.19]	−0.99 (1.27) [−4–1.33]	< .001
Visuospatial Functioning composite	−0.4 (0.77) [−3.5 –1.14]	−0.14 (0.75) [−3.5–1.14]	−0.64 (0.71) [−3.5–0.96]	< .001
Language composite	0.29 (0.88) [−2.7–1.78]	0.5 (0.7) [−1.28–1.78]	0.09 (0.98) [−2.7–1.47]	.002

Data are mean and standard deviation unless indicated otherwise. *P*-values of independent sample *t*-tests or *χ*^2^-tests between individuals with PD-N and PD-MCI are presented. The cognitive diagnosis (PD-N and PD-MCI) refers to the LEQ baseline (not the DEMPARK/LANDSCAPE baseline). CERAD-Plus Total Score, Consortium to establish a Registry of Alzheimer’s Disease Plus Total Score; F, female; GDS, Geriatric Depression Scale; LEDD, Levodopa Equivalent Daily Dose; LEQ, Lifetime of Experiences Questionnaire; M: male; MMSE, Mini-Mental State Examination; PANDA, Parkinson Neuropsychometric Dementia Assessment; PD-MCI, Parkinson’s disease with mild cognitive impairment; PD-N, Parkinson’s disease without cognitive impairment; UPDRS-III, Unified Parkinson´s Disease Rating Scale Part 3

Sixty-two percent (*n* = 105) of the individuals did not complete their last possible follow-up (3- or 4 years, depending on the LEQ baseline). Individuals lost to attrition at their maximum possible follow-up were significantly older at LEQ baseline (71.52 ± 6.23 years) than individuals who completed their last possible follow-up (69.28 ± 6.73 years), *t*(167) = 2.20, *p* = 0.029. Importantly, there were no significant differences regarding clinical and neuropsychological parameters. Further details are displayed in Supplementary Table S2.

### Cognitive status and cognitive reserve

**Fig. 2:**
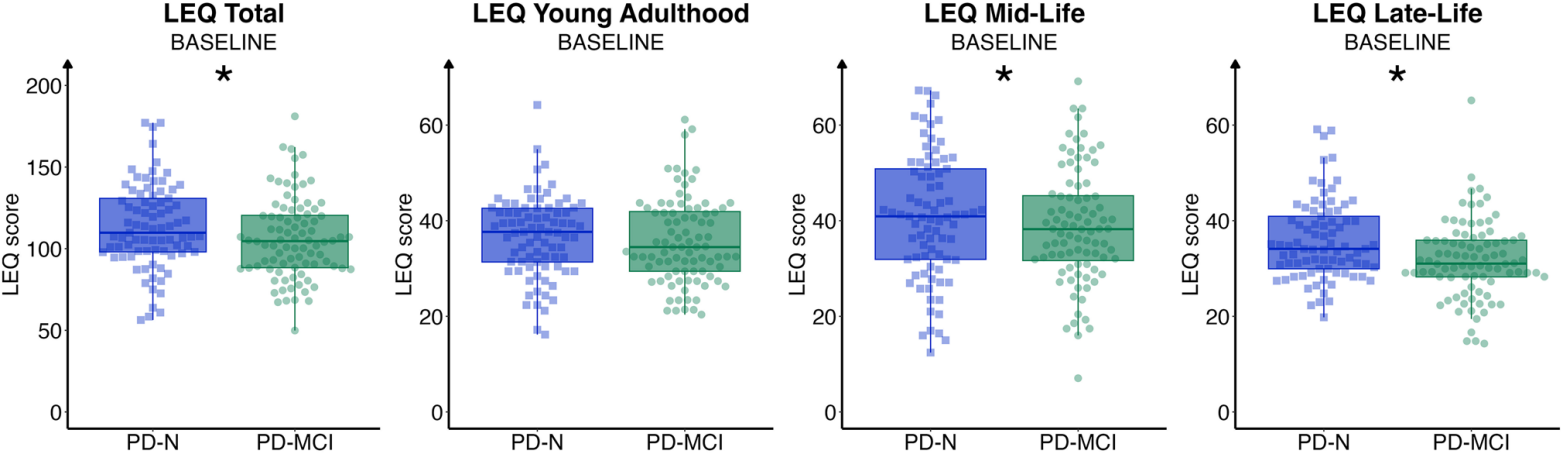
Baseline scores of the Lifetime of Experiences Questionnaire by diagnostic category. Baseline scores of the Lifetime of Experiences Questionnaire (LEQ) by diagnostic category, i.e., between individuals with Parkinson’s disease without cognitive impairment (PD–N, squares) and individuals with mild cognitive impairment (PD-MCI, dots) at the corresponding LEQ baseline. Dots/squares represent individual LEQ (sub-)scores, the group-wise boxplots visualize the within-group median, the hinges represent the corresponding first and the third quartile, and the whiskers are 1.5 times the inter-quartile range. *p < 0.050.

Controlled for age, sex, UPDRS-III, and disease duration, individuals with PD-N had significantly higher LEQ total scores compared to individuals with PD-MCI [*F*(1,153) = 4.609, *p* = 0.033, *η*_p_^2^ = 0.029] with a small effect size. Additionally, individuals with PD-N had significantly higher mid-life [*F*(1,153) = 4.724, *p* = 0.031, *η*_p_^2^ = 0.030] and late-life [*F*(1,153) = 6.23, *p* = 0.014, *η*_p_^2^ = 0.039] LEQ subscores, both with small effect sizes. No meaningful group difference in the young adulthood LEQ subscore was observed [*F*(1,153) = 0.433, *p* = 0.511, *η*_p_^2^ = 0.003]. The distribution of LEQ scores across cognitive diagnoses is displayed in Fig. 2.

### Cognitive performance and cognitive reserve

The adjusted *R*^2^ (_adj_*R*^2^) of the evaluated multiple linear regression models (Table 2) ranged between 0.16 (CERAD-Plus total score) and 0.27 (executive composite score). However, since the model for the language composite score was not significant [*F*(8,147) = 0.88, *p* = 0.536, _adj_*R*^2^=0.01], it was not further interpreted.

**Table 2:** Multiple linear regression models evaluating the influence of cognitive reserve of different life-stages on cognitive performance

	Predictors								
	Intercept	LEQ Young Adulthood	LEQ Mid-Life	LEQ Late-Life	Age	Sex: Female	UPDRS-III	Disease Duration	Depressive Symptoms
CERAD-Plus Total Score	0.293 (0.01 – 0.575) *p* = .044	−0.014 (−0.029; – 0.002) *β* = −0.185 *p* = .083	0.013 (0.002; – 0.024) *β* = 0.251 *p* = .023	0.015 (0.001; – 0.029) *β* = 0.2 *p* = .043	−0.015 (−0.03; – 0) *β* = −0.154 *p* = .046	0.223 (0.001; – 0.445) *β* = 0.157 *p* = .051	−0.011 (−0.02 to −0.003) *β* = − 0.21 *p* = .008	0 (−0.002; – 0.002) *β* = 0.025 *p* = .743	−0.029 (−0.066; – 0.008) *β* = −0.128 *p* = .128
	*F*(8,148) = 4.71, *p* < .001, _adj_*R*^2^ = 0.16								
Memory	−0.109 (−0.459; – 0.24) *p* = .541	0.007 (−0.012; – 0.026) *β* = 0.072 *p* = .474	0.015 (0.001;–0.028) *Β* = 0.224 *p* = .032	0.006 (−0.012; – 0.024) *β* = 0.065 *p* = .491	−0.047 (−0.065 to −0.028) *β* = −0.361 *p* < .001	0.395 (0.121; – 0.67) *β* = 0.215 *p* = .005	−0.008 (−0.018; – 0.003) *β* = −0.108 *p* = .149	−0.001 (−0.003; – 0.002) *β* = −0.035 *p* = .623	−0.056 (−0.102 to −0.01) *β* = −0.191 *p* = .018
	*F*(8,148) = 6.98, *p* < .001, _adj_*R*^2^ = 0.23								
Executive Functions	−0.768 (−1.096 to −0.441) *p* < .001	0.004 (−0.014; – 0.022) *β* = 0.041 *p* = .679	0.014 (0.002; – 0.027) *β* = 0.227 *p* = .026	0.017 (0 – 0.034) *β* = 0.183 *p* = .047	−0.055 (−0.072 to −0.037) *β* = −0.442 *p* < .001	0.461 (0.204; – 0.719) *β* = 0.261 *p* = .001	0 (−0.01; – 0.009) *β* = −0.003 *p* = .969	0.001 (−0.002; – 0.003) *β* = 0.035 *p* = .618	−0.021 (−0.064; – 0.022) *β* = −0.074 *p* = .343
	*F*(8,148) = 8.31, *p* < .001, _adj_*R*^2^ = 0.27								
Attention	−0.061 (−0.556; – 0.434) *p* = .810	− 0.001 (−0.029; – 0.026) *β* = −0.011 *p* = .915	0.011 (−0.008; – 0.03) *β* = 0.117 *p* = .262	0.023 (−0.002; – 0.049) *β* = 0.171 *p* = .072	−0.066 (−0.092 to −0.039) *β* = −0.359 *p* < .001	0.513 (0.12; – 0.906) *β* = 0.195 *p* = .012	−0.021 (−0.036 to −0.007) *β* = −0.214 *p* = .005	0 (−0.003; – 0.004) *β* = 0.014 *p* = .842	−0.06 (−0.125; – 0.005) *β* = −0.146 *p* = .071
	*F*(8,146) = 7.1, *p* < .001, _adj_*R*^2^ = 0.24								
Visuospatial Functioning	0.097 (−0.24; – 0.434) *p* = .573	0.001 (−0.018; – 0.019) *β* = 0.006 *p* = .955	0.018 (0.005; – 0.031) *β* = 0.292 *p* = .008	0.001 (−0.017; – 0.018) *β* = 0.008 *p* = .936	−0.034 (−0.052 to −0.016) *β* = −0.286 *p* < .001	0.014 (−0.252; – 0.28) *β* = 0.008 *p* = .920	−0.018 (−0.028 to −0.008) *β* = −0.272 *p* = .001	0 (−0.003; – 0.002) *β* = −0.029 *p* = .694	−0.009 (−0.053; – 0.036) *β* = −0.032 *p* = .703
	*F*(8,146) = 5.61, *p* < .001, _adj_*R*^2^ = 0.19								
Language	0.256 (−0.174; – 0.685) *p* = .245	−0.007 (−0.03; – 0.017) *β* = −0.065 *p* = .573	0 (−0.016; – 0.017) *β* = 0.005 *p* = .966	0.024 (0.001; – 0.046) *β* = 0.224 *p* = .039	−0.003 (−0.026; – 0.02) *β* = −0.024 *p* = .780	0.164 (−0.174; – 0.501) *β* = 0.083 *p* = .343	0 (−0.012; – 0.013) *β* = 0.001 *p* = .986	−0.001 (−0.004; – 0.002) *β* = −0.072 *p* = .385	0.026 (−0.031; – 0.082) *β* = 0.083 *p* = .371
	*F*(8,147) = 0.88, *p* = .536, _adj_*R*^2^ = − 0.01								

*Notes.* Data are unstandardized coefficients and (95% confidence intervals). Furthermore, standardized *β* coefficients and corresponding *p*-values are reported. LEQ subscores and age were mean-centered prior to model estimation. LEQ, Lifetime of Experiences Questionnaire; UPDRS-III, Unified Parkinson’s Disease Rating Scale Part 3

The young adulthood LEQ subscore was not found to be a meaningful independent determinant of cognitive performance, neither for overall cognitive function nor any specific domain. The mid-life LEQ subscore was found to be a significant independent determinant of cognitive performance in overall cognitive function, executive functions, memory, and visuospatial functions. Similarly, the late-life LEQ subscore was identified as a significant independent determinant of cognitive performance in overall cognitive function and executive functions. Higher LEQ subscores during these life-stages were positively correlated with better cognitive performance in the mentioned cognitive domains. The strength of these associations was small (0.2 ≤ $\left| \beta \right|$ ≤ 0.292) with a tendency of the mid-life LEQ score being stronger associated with cognitive performance than the late-life LEQ score.

Beyond the LEQ scores, age was found to be a significant independent determinant of cognitive performance for overall cognitive function and all cognitive domains, indicating that younger age was associated with better cognitive performance with small to moderate effect sizes (0.157 ≤ $\left| \beta \right|$ ≤ 0.442). Female sex was associated with better cognitive performance in the domains of memory, executive functions, and attention, with small effect sizes (0.157 ≤ $\left| \beta \right|$ ≤ 0.261). Moreover, motor symptom severity was determined to be a significant determinant of cognitive performance for overall cognitive function, attention, and visuospatial functions. Here, higher motor symptom severity was associated with poorer cognitive performance with small effect sizes (0.210 ≤ $\left| \beta \right|$ ≤ 0.272). More depressive symptoms were significantly associated with poorer cognitive performance in the domain of memory with a small effect size ($\left| \beta \right|$= 0.191). Disease duration was not found to be a meaningful independent determinant of cognitive performance.

### Cognitive trajectories and cognitive reserve

For the LME models on cognitive trajectories over time (Table 3), marginal *R*^2^ ranges between 0.236 (CERAD-Plus total score) and 0.441 (executive functions) and conditional *R*^2^ ranges between 0.754 (executive functions) and 0.880 (attention). The standardized *β* coefficients indicate small effect sizes for sex, disease duration, motor symptom severity and depressive symptoms, and small to moderate effect sizes for age and the cognitive diagnostic category.

**Table 3:** Linear mixed effect models evaluating the influence of cognitive reserve on cognitive performance over time (max. 4-year follow-up)

Fixed effects	CERAD-Plus Total Score			Executive Functions			Memory			Attention			Visuospatial Functioning		
	Estimates	*β*	*p*	Estimates	*β*	*p*	Estimates	*β*	*p*	Estimates	*β*	*p*	Estimates	*β*	*p*
Intercept	0.531 (0.264; – 0.798)		** < .001**	−0.426 (−0.711 to −0.142)		**.003**	0.112 (−0.211; – 0.435)		.496	0.302 (−0.199; – 0.802)		.237	0.237 (−0.092; – 0.566)		.158
Time	−0.095 (−0.131 to −0.059)	−0.190	** < .001**	− 0.140 (−0.179 to −0.101)	−0.210	** < .001**	−0.054 (−0.101 to −0.007)	−0.081	**.024**	−0.259 (−0.328 to −0.189)	−0.247	** < .001**	−0.096 (−0.136 to −0.057)	−0.147	** < .001**
LEQ total	0.004 (0.001; – 0.007)	0.136	**.036**	0.009 (0.005; – 0.013)	0.271	** < .001**	0.008 (0.004; – 0.013)	0.248	** < .001**	0.008 (0.001; – 0.015)	0.157	**.019**	0.008 (0.003; – 0.012)	0.229	**.002**
Time*LEQ Total	−0.002 (−0.003 to −0.000)	−0.120	**.034**	− 0.001 (−0.003; – 0.000)	−0.072	.115	−0.002 (−0.004 to −0.000)	−0.109	**.049**	−0.001 (−0.004; – 0.002)	−0.030	.560	−0.001 (−0.002; – 0.001)	−0.041	.384
Age	−0.006 (−0.020; – 0.007)	−0.064	.358	− 0.041 (−0.055 to − 0.027)	−0.323	** < .001**	−0.040 (−0.056 to −0.024)	−0.314	** < .001**	−0.056 (−0.082 to −0.031)	−0.281	** < .001**	−0.030 (−0.047 to −0.014)	−0.239	** < .001**
Sex: female	0.091 (−0.111; – 0.292)	0.063	.377	0.232 (0.022; – 0.442)	0.124	**.031**	0.208 (−0.030; – 0.447)	0.112	.087	0.309 (− 0.074; – 0.692)	0.105	.114	−0.204 (−0.450; – 0.042)	−0.110	.104
Diagnosis: PD-MCI	−0.511 (−0.692 to −0.331)	−0.392	** < .001**	−0.773 (−0.961 to − 0.584)	−0.454	** < .001**	−0.480 (−0.694 to −0.266)	−0.284	** < .001**	−0.649 (−0.990 to −0.308)	−0.241	** < .001**	−0.394 (−0.615 to −0.173)	−0.235	** < .001**
Disease Duration	−0.000 (−0.002; – 0.002)	−0.007	.915	0.000 (−0.002; – 0.002)	0.018	.719	−0.001 (−0.003; – 0.001)	−0.069	.239	0.000 (−0.003; – 0.004)	0.010	.862	−0.001 (−0.003; – 0.001)	−0.041	.507
UPDRS-III	−0.007 (−0.015; – 0.001)	−0.119	.067	0.005 (−0.003; – 0.013)	0.069	.194	−0.006 (−0.015; – 0.003)	−0.074	.219	−0.018 (−0.032 to −0.004)	−0.147	**.013**	−0.014 (−0.024 to −0.005)	−0.189	**.003**
Depressive Symptoms	−0.026 (−0.059; – 0.006)	−0.111	.115	−0.017 (−0.051; – 0.017)	−0.056	.326	−0.029 (−0.067; – 0.010)	−0.094	.146	−0.059 (−0.120; – 0.003)	−0.122	.062	0.005 (−0.035; – 0.045)	0.016	.814
Random Effects															
σ^2^	0.08			0.18			0.17			0.24			0.15		
τ_00_	0.23 _ID_			0.22 _ID_			0.34 _ID_			0.83 _ID_			0.35 _ID_		
τ_11_	0.02 _ID.time_			0.01 _ID.time_			0.03 _ID.time_			0.09 _ID.time_			0.02 _ID.time_		
ρ_01_	−0.03 _ID_			0.24 _ID_			−0.21 _ID_			0.15 _ID_			−0.09 _ID_		
ICC	0.79			0.56			0.70			0.84			0.73		
N	157 _ID_			157 _ID_			157 _ID_			157 _ID_			156 _ID_		
Observations	474			496			497			480			502		
Marginal *R*^2^ / Conditional *R*^2^	0.236 / 0.836			0.441 / 0.754			0.266 / 0.781			0.251 / 0.880			0.249 / 0.796		

*Notes.* Data are unstandardized coefficients and (95% confidence intervals). Furthermore, standardized *β* coefficients and corresponding *p*-values are reported. LEQ subscores and age were mean-centered prior to model estimation. ICC, intraclass correlation coefficient, LEQ, Lifetime of Experiences Questionnaire; UPDRS-III, Unified Parkinson’s Disease Rating Scale Part 3; σ^2^, variance of residual errors; τ_00_, variance of the random intercepts; τ_11_, variance of the random slopes; ρ_01_, covariance between random intercepts and random slopes

**Fig. 3:**
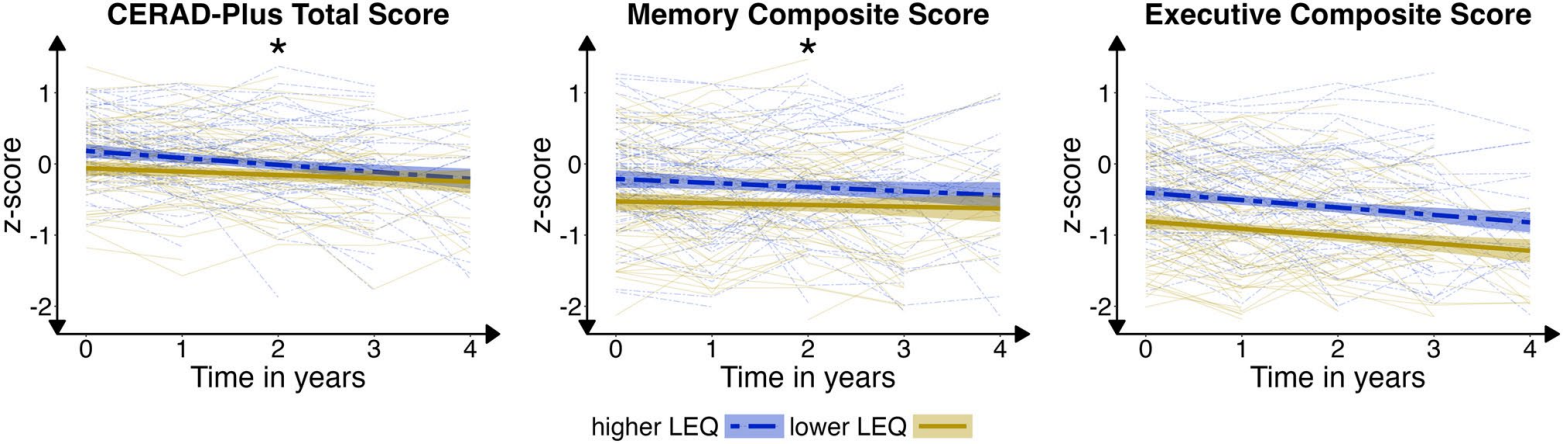
Cognitive Trajectories Over Time. Observed and predicted cognitive trajectories for individuals with higher vs. lower scores (determined by median split, for visualization only) in the Lifetime of Experiences Questionnaire for the CERAD-Plus total score and the memory composite score (both of which show the significant interaction between time*LEQ), and the executive composite score (as an example of stable effects of cognitive reserve over time)

Time emerged as a significant independent negative determinant of cognitive performance across all cognitive outcomes, indicating a decline in cognitive performance with increasing time since baseline assessment. The LEQ total score was found to be a significant independent positive determinant of cognitive performance across all cognitive outcomes, suggesting that higher LEQ scores were associated with better cognitive performance with a small effect size. Although differences were small, the time*LEQ interaction was found to be significantly negative for the CERAD-Plus total score and the memory composite score only, indicating that the decline in cognitive performance over time tended to be stronger among individuals with higher LEQ scores. There was no meaningful time*LEQ interaction for executive functions, attention, and visuospatial functioning. Figure 3 presents both the observed individual cognitive trajectories and the mean predicted trajectories for individuals categorized into higher vs. lower LEQ scores.

The life-stage specific vs. non-specific associations with cognitive performance over time are explored in the Supplementary Material including Supplementary Figure S2. Four-year follow-up data was only available for *n* = 39 (out of *n* = 169 at LEQ baseline). To check the robustness of results, the longitudinal analyses were repeated until the 3-year follow-up only. The marginal *R*^2^ ranges between 0.244 (CERAD-Plus total score) and 0.408 (executive functions) and conditional *R*^2^ ranges between 0.791 (visuospatial functions) and 0.897 (attention). The effects of Time and LEQ total score appeared robust, however, the time*LEQ interaction was significant for the memory composite only. The full LME models with data until the 3-year follow-up only are reported in Supplementary Table S3.

## Discussion

The aim of this study was to evaluate the association of a multidimensional proxy of CR, the LEQ [18], which quantifies the complexity of lifestyle activities, with the presence of cognitive impairment and cognitive functioning across multiple cognitive domains in individuals with PD both cross-sectionally and longitudinally. For this purpose, we used data from the DEMPARK/LANDSCAPE study [21].

We found that (i) individuals with PD-N had slightly higher CR compared to those with PD-MCI; (ii) A higher CR, particularly as reported during the stages of mid- and late-life, was associated with slightly better overall cognitive performance and performance in executive functions, memory, attention, and visuospatial functioning; (iii) Individuals with higher CR demonstrated slightly better cognitive functioning across time. Notably, with higher levels of CR, the protective effect of CR tended to diminish in global cognitive functioning and memory, while no accelerated cognitive decline with higher levels of CR was observed in the other domains.

Our findings are in line with previous research on CR in individuals with PD, which has consistently shown a positive association between levels of CR and cognitive status [13] as well as cognitive performance [11–13, 15, 20]. In the present study, the use of the LEQ subscores allowed us to assess the influence of CR at different life-stages on cognitive status. The use of life-stage specific proxies is important not only for determining which specific lifestyle actions are relevant for preserving cognitive health and when to start with prevention and intervention strategies against cognitive decline, but also for critically reflecting on the possible bidirectional association between CR and cognitive performance [4]. It promotes the understanding of whether CR acts as a protective factor against cognitive decline or if prodromal or apparent cognitive decline rather leads to less engagement in complex lifestyle activities [4, 20].

### Cognitive reserve and reverse causation

The phenomenon of reverse causation constitutes a major limitation in CR research in general. We cannot rule out the possibility that the observed differences in CR, particularly in the late-life stage, between the two diagnostic subgroups PD-N and PD-MCI are influenced in a reverse causal manner. Particularly those with PD-MCI may have already reduced their lifestyle activities not only recently but also in the prodromal phase of this cognitive decline and the prodromal phase of PD in general. It has recently been shown that lower CR operationalized by the Cognitive Reserve Index questionnaire [35] was associated with worse cognitive performance and higher rates of MCI in individuals with isolated REM sleep behavior disorder [36], a possible prodromal phase of PD. Furthermore, the findings of Kremen, et al. [14] emphasize that our reported effects of mid- and late-life CR proxies may not be truly causal but instead reflect top-down effects of general intellectual capacity assessed early in life [14, 37].

Notably, we observed domain-specific effects of life-stage specific CR: An independent association between CR proxies from both mid-life and late-life was found with global cognition and executive functions. For memory and visuospatial functioning, only the CR proxy of mid-life was positively associated with cognitive performance. The domain-specific findings observed in our cross-sectional analyses align with the hypothesis that CR has stronger associations with cognitive performance in domains that are more susceptible to decline in PD, i.e., executive functions [11, 12, 15]. These findings, however, will also be augmented by reverse causal mechanisms, thereby artificially amplifying the association between CR and cognition [4, 14]. Even the LEQ mid-life score (30–65 years) will be influenced by reverse causation as it includes the prodromal phase of PD and cognitive decline associated with PD.

Since it is not conceivable or possible to randomly control the access to primary and secondary education, job complexity and cognitively stimulating lifestyle activities on a study design level, we can only statistically control for these variables. We consistently found a positive association between the LEQ total score and cognitive performance across time, even when we controlled for the cognitive diagnosis (PD-N vs. PD-MCI) in the longitudinal analyses, pointing towards the protective effect of cognitive reserve despite the presence of reverse causal mechanisms. Future analytical approaches may also include cross-lagged panel models, allowing to investigate the causal direction of the relation of two variables over time if time-lagged data of both variables is available. The application of such models in the context of cognitive reserve reveals the expected bidirectional relationship with an emphasis on the protective role of lifestyle choices compared to reverse causal mechanisms [38, 39].

Beyond these statistical approaches, some promising findings on the potentially disease-modifying effects of lifestyle interventions for slowing down cognitive decline in the context of Alzheimer’s disease especially for those at risk [40] and single-domain intervention studies (e.g., on cognitive training) in PD [41], provide evidence for the bidirectional association between CR and cognitive performance. To summarize: In the absence of necessary information and perfect study designs, the most realistic approach is to acknowledge the presence of bidirectional influences and to be mindful of the potential of overestimated effects of mid- and late-life complex lifestyle activities. While many studies on CR neglect or do not even mention reverse causation, the proactive discussion of potential confounders should be an integral part of any publication investigating the role of CR.

### Domain-specificity and cognitive trajectories

We further examined the domain-specific effects of CR longitudinally. Overall, we observed a decline in cognitive performance across all domains as time progressed. CR emerged as a significant independent protective factor for cognitive performance: irrespective of time, across all cognitive domains. Notably, the largest protective effect of CR was observed in the domain of executive functions. The observed associations between CR proxies and all covariates were small and overall smaller than the association of age with cognitive performance, which is well in line with previous CR research [19].

Previous research has consistently shown that individuals with PD and higher CR experience less decline or have a lower risk of developing cognitive dysfunction [8, 20, 42]. Guzzetti, et al. [11] proposed that the protective effect of CR increases over the course of PD until a point of inflection is reached. At this point, neuropathology overwhelms the compensatory processes associated with CR, leading to a steeper decline in cognitive performance, which can be interpreted as a “terminal drop” [11]. In contrast, in AD, differences in cognitive performance between individuals with higher and lower CR tend to diminish shortly after the clinical presentation of the disease, i.e., the point of inflection is reached earlier in AD compared to PD, due to the disease-defining nature of cognitive impairment in AD compared to PD [6, 11].

Our findings in PD align with this hypothesis of a general protective effect of CR and a “terminal drop” trajectory of cognitive performance for individuals with higher CR. However, our data rather indicate domain-specific effects of CR instead of disease-general mechanisms. We observed that individuals with PD and higher CR exhibited a steeper decline in memory and overall cognitive functioning compared to those with lower CR. This pattern of decline in the memory domain and the CERAD-Plus total score [29] in our PD sample aligns with the postulated AD-trajectories of cognitive decline as proposed by Guzzetti, et al. [11]. Even though the CERAD-Plus total score was treated as a proxy for global cognition, the composite score is still memory-dominated [29]. Therefore, the observed pattern for the CERAD-Plus total score may be skewed by the memory contribution. Notably, the domains of executive functions, attention, and visuospatial functioning follow the proposed PD-trajectories of cognitive functioning with a delayed terminal drop. This strengthens the cross-sectional findings of a slightly more pronounced effect of CR in vulnerable cognitive domains in PD, including executive functions [11, 12, 15]. This is especially interesting as in individuals with isolated REM sleep behavior disorder, who may already show cognitive alterations in various cognitive domains, e.g., executive functions [43, 44], CR may modulate the development of cognitive changes and the transition to motor-dominant α-synucleinopathies [36], highlighting the potential of CR as a truly protective marker for neurodegeneration. Some studies even suggest a beneficial effect of CR on motor outcomes in PD [11, 45]. More research on CR in prodromal disease phases is desired.

### Strengths and limitations

With a database comprising 169 individuals with PD across different levels of cognitive impairment in the cross-sectional database and more than 500 observations for the longitudinal analyses with up to 4-year follow-up data, the study outperforms the majority of available studies in the field in terms of sample size and the number of available longitudinal observations over a significant time period [42]. However, 62% of the individuals did not complete their last possible follow-up, which constitutes a higher attrition rate compared to other longitudinal studies in PD. The nationwide data collection is a strength of the DEMPARK/LANDSCAPE database.

The comprehensive cognitive assessment battery enabled examination of domain-specific effects, moving beyond a sole focus on global cognition. One limitation is that the cross-sectional comparison of CR between PD-N and PD-MCI relies on the PD-MCI criteria available at the time of initial set-up, which have since become revised and may overestimate the presence of MCI [31]. Beyond the already discussed strengths and limitations associated with the use of the LEQ [18], a multidimensional proxy of CR, which accounted for complex lifestyle activities across different life stages, no proxy of socio-economic status [9, 46] was available.

The current analysis focused on selected neuropsychological, demographic and clinical parameters to predict cognitive decline and to model the association between CR and cognitive performance. This neglects the potential contribution of several other parameters potentially associated with cognitive decline in PD as reviewed in Aarsland, et al. [1], such as genetic subtypes (e.g., mutations in the β-glucocerebrosidase gene, GBA) [47], subtypes regarding the onset of PD (early vs. late) [48], changes in medication (especially anticholinergic drugs) [49, 50], and direct brain imaging correlates. A subgroup of the sample of the current analyses underwent magnetic resonance imaging (MRI) at their LEQ baseline (*n* = 67, 39.6%), which may allow for further analyses combining neuropsychological, demographic, clinical, and brain imaging parameters. For the present longitudinal analyses, however, the LEQ + MRI sample would have been too small. To fully understand the contributing factors to cognitive decline in PD, multimodal prediction models effectively combining different types of data (such as neuropsychological, demographic, clinical, brain imaging, genetic assessments) with advanced, such as artificial intelligence-assisted techniques for feature selection, may be beneficial. Another limitation is that the full impact of CR on cognitive trajectories may require longer follow-up periods to be fully understood.

## Conclusion

Overall, our data suggest that CR, operationalized by complex lifestyle activities across the lifespan, especially in mid- and late-life, appears to be associated with cognitive functioning in individuals with PD. Furthermore, its influence varies across different cognitive domains: While CR seems to be protective for all cognitive domains, it is also related to a slightly more pronounced drop of functioning for global cognition and memory. These results contribute to our understanding of the factors influencing cognitive decline in PD and have implications for developing targeted interventions and prevention strategies to optimize cognitive functioning in individuals with PD.

### Supplementary Information

Below is the link to the electronic supplementary material.

Supplementary file1 (PDF 930 KB)

## Data Availability

The data included in this study are available upon reasonable request from the corresponding author.

## References

[1] Aarsland D, Batzu L, Halliday GM, Geurtsen GJ, Ballard C, Ray Chaudhuri K, Weintraub D (2021) Parkinson disease-associated cognitive impairment. Nat Rev Dis Primers 7:1–21 34210995 10.1038/s41572-021-00280-3

[2] Baiano C, Barone P, Trojano L, Santangelo G (2020) Prevalence and clinical aspects of mild cognitive impairment in parkinson’s disease: a meta-analysis. Mov Disord 35:45–54 31743500 10.1002/mds.27902

[3] Saredakis D, Collins-Praino LE, Gutteridge DS, Stephan BC, Keage HA (2019) Conversion to MCI and dementia in Parkinson’s disease: a systematic review and meta-analysis. Parkinsonism Relat Disord 65:20–31 31109727 10.1016/j.parkreldis.2019.04.020

[4] Stern Y, Arenaza-Urquijo EM, Bartrés-Faz D, Belleville S, Cantilon M, Chetelat G, Ewers M, Franzmeier N, Kempermann G, Kremen WS (2020) Whitepaper: defining and investigating cognitive reserve, brain reserve, and brain maintenance. Alzheimers Dement 16:1305–1311 30222945 10.1016/j.jalz.2018.07.219 PMC6417987

[5] Stern Y, Barnes CA, Grady C, Jones RN, Raz N (2019) Brain reserve, cognitive reserve, compensation, and maintenance: operationalization, validity, and mechanisms of cognitive resilience. Neurobiol Aging 83:124–129 31732015 10.1016/j.neurobiolaging.2019.03.022 PMC6859943

[6] Stern Y (2012) Cognitive reserve in ageing and Alzheimer’s disease. Lancet Neurol 11:1006–1012 23079557 10.1016/S1474-4422(12)70191-6 PMC3507991

[7] Nelson ME, Jester DJ, Petkus AJ, Andel R (2021) Cognitive reserve, Alzheimer’s neuropathology, and risk of dementia: a systematic review and meta-analysis. Neuropsychol Rev 31:233–250 33415533 10.1007/s11065-021-09478-4 PMC7790730

[8] Gu L, Xu H (2022) Effect of cognitive reserve on cognitive function in Parkinson’s disease. Neurol Sci 43:4185–4192 35230598 10.1007/s10072-022-05985-1

[9] Pinto JO, Peixoto B, Dores AR, Barbosa F (2023) Measures of cognitive reserve: an umbrella review. Clin Neuropsychol 38(1):42–115. 10.1080/13854046.2023.2200978 37073431 10.1080/13854046.2023.2200978

[10] Kartschmit N, Mikolajczyk R, Schubert T, Lacruz ME (2019) Measuring cognitive reserve (CR)—a systematic review of measurement properties of CR questionnaires for the adult population. PLoS ONE 14:e0219851 31390344 10.1371/journal.pone.0219851 PMC6685632

[11] Guzzetti S, Mancini F, Caporali A, Manfredi L, Daini R (2019) The association of cognitive reserve with motor and cognitive functions for different stages of Parkinson’s disease. Exp Gerontol 115:79–87 30502539 10.1016/j.exger.2018.11.020

[12] Ciccarelli N, Monaco MRL, Fusco D, Vetrano DL, Zuccalà G, Bernabei R, Brandi V, Pisciotta MS, Silveri MC (2018) The role of cognitive reserve in cognitive aging: what we can learn from Parkinson’s disease. Aging Clin Exp Res 30:877–880 29019160 10.1007/s40520-017-0838-0

[13] Ciccarelli N, Colombo B, Pepe F, Magni E, Antonietti A, Silveri MC (2022) Cognitive reserve: a multidimensional protective factor in Parkinson’s disease related cognitive impairment. Aging Neuropsychol Cogn 29:687–702 10.1080/13825585.2021.1892026 33629649

[14] Kremen WS, Elman JA, Panizzon MS, Eglit GM, Sanderson-Cimino M, Williams ME, Lyons MJ, Franz CE (2022) Cognitive reserve and related constructs: a unified framework across cognitive and brain dimensions of aging. Front Aging Neurosci 14:834765 35711905 10.3389/fnagi.2022.834765 PMC9196190

[15] Loftus AM, Gasson N, Lopez N, Sellner M, Reid C, Cocks N, Lawrence BJ (2021) Cognitive reserve, executive function, and memory in parkinson’s disease. Brain Sci 11:992 34439609 10.3390/brainsci11080992 PMC8391924

[16] Kalbe E, Rehberg SP, Heber I, Kronenbuerger M, Schulz JB, Storch A, Linse K, Schneider C, Gräber S, Liepelt-Scarfone I (2016) Subtypes of mild cognitive impairment in patients with Parkinson’s disease: evidence from the LANDSCAPE study. J Neurol Neurosurg Psychiatry 87:1099–1105 27401782 10.1136/jnnp-2016-313838

[17] Kehagia AA, Barker RA, Robbins TW (2013) Cognitive impairment in Parkinson’s disease: The dual syndrome hypothesis. Neurodegener Dis 11:79–92 23038420 10.1159/000341998 PMC5079071

[18] Valenzuela MJ, Sachdev P (2007) Assessment of complex mental activity across the lifespan: development of the lifetime of experiences questionnaire (LEQ). Psychol Med 37:1015–1025 17112402 10.1017/S003329170600938X

[19] Hindle JV, Martin-Forbes PA, Martyr A, Bastable AJ, Pye KL, Mueller Gathercole VC, Thomas EM, Clare L (2017) The effects of lifelong cognitive lifestyle on executive function in older people with Parkinson’s disease. Int J Geriatr Psychiatry 32:e157–e165 28170111 10.1002/gps.4677

[20] Hindle JV, Hurt CS, Burn DJ, Brown RG, Samuel M, Wilson KC, Clare L (2016) The effects of cognitive reserve and lifestyle on cognition and dementia in Parkinson’s disease—a longitudinal cohort study. Int J Geriatr Psychiatry 31:13–23 25781584 10.1002/gps.4284

[21] Balzer-Geldsetzer M, Da Costa ASFB, Kronenbürger M, Schulz JB, Röske S, Spottke A, Wüllner U, Klockgether T, Storch A, Schneider C (2011) Parkinson’s disease and dementia: a longitudinal study (DEMPARK). Neuroepidemiology 37:168–176 22067139 10.1159/000331490

[22] Hughes AJ, Daniel SE, Kilford L, Lees AJ (1992) Accuracy of clinical diagnosis of idiopathic Parkinson’s disease: a clinico-pathological study of 100 cases. J Neurol Neurosurg Psychiatry 55:181–184 1564476 10.1136/jnnp.55.3.181 PMC1014720

[23] Emre M (2003) Dementia associated with Parkinson’s Disease. Lancet Neurol 2:229–237 12849211 10.1016/s1474-4422(03)00351-x

[24] Tomlinson CL, Stowe R, Patel S, Rick C, Gray R, Clarke CE (2010) Systematic review of levodopa dose equivalency reporting in Parkinson’s Disease. Mov Disord 25:2649–2653 21069833 10.1002/mds.23429

[25] Fahn S (1987) Unified Parkinson’s disease rating scale. In: Fahn SMC, Goldstein M, Calne DB (eds) Recent developments in Parkinson’s Disease. Macmillan Healthcare Information, Florham Park, pp 153–163

[26] Yesavage JA, Brink TL, Rose TL, Lum O, Huang V, Adey M, Leirer VO (1983) Development and validation of a geriatric depression screening scale: a preliminary report. J Psychiatr Res 17:37–49 10.1016/0022-3956(82)90033-4 7183759

[27] Folstein MF, Folstein SE, McHugh PR (1975) “Mini-mental state”: a practical method for grading the cognitive state of patients for the clinician. J Psychiatr Res 12:189–198 1202204 10.1016/0022-3956(75)90026-6

[28] Kalbe E, Calabrese P, Kohn N, Hilker R, Riedel O, Wittchen H-U, Dodel R, Otto J, Ebersbach G, Kessler J (2008) Screening for cognitive deficits in Parkinson’s disease with the Parkinson neuropsychometric dementia assessment (PANDA) instrument. Parkinsonism Relat Disord 14:93–101 17707678 10.1016/j.parkreldis.2007.06.008

[29] Lillig R, Ophey A, Schulz JB, Reetz K, Wojtala J, Storch A, Liepelt-Scarfone I, Becker S, Berg D, Balzer-Geldsetzer M (2021) A new CERAD total score with equally weighted z-scores and additional executive and non-amnestic „CERAD-Plus “tests enhances cognitive diagnosis in patients with Parkinson’s disease: evidence from the LANDSCAPE study. Parkinsonism Relat Disord 90:90–97 34418761 10.1016/j.parkreldis.2021.07.034

[30] Aebi C (2002) Validierung der neuropsychologischen Testbatterie CERAD-NP: eine Multi-Center Studie. In:University of Basel, Basel

[31] Petersen RC (2004) Mild cognitive impairment as a diagnostic entity. J Intern Med 256:183–194 15324362 10.1111/j.1365-2796.2004.01388.x

[32] Ourry V, Marchant NL, Schild A-K, Coll-Padros N, Klimecki OM, Krolak-Salmon P, Goldet K, Reyrolle L, Bachelet R, Sannemann L (2021) Harmonisation and between-country differences of the lifetime of experiences questionnaire in older adults. Front Aging Neurosci 13:740005 34720992 10.3389/fnagi.2021.740005 PMC8551756

[33] R Core Team (2022) R: A language and environment for statistical computing. R Foundation for Statistical Computing, Vienna, Austria. URL https://www.R-project.org/.In:

[34] Bates D, Mächler M, Bolker B, Walker S (2015) Fitting linear mixed-effects models using lme4. J Stat Softw 67:1–48

[35] Nucci M, Mapelli D, Mondini S (2012) Cognitive reserve index questionnaire (CRIq): a new instrument for measuring cognitive reserve. Aging Clin Exp Res 24:218–226 21691143 10.3275/7800

[36] D’Este G, Berra F, Carli G, Leitner C, Marelli S, Zucconi M, Casoni F, Ferini-Strambi L, Galbiati A (2023) Cognitive reserve in isolated rapid eye-movement sleep behavior disorder. Brain Sci 13:176 36831719 10.3390/brainsci13020176 PMC9954116

[37] Kremen WS, Beck A, Elman JA, Gustavson DE, Reynolds CA, Tu XM, Sanderson-Cimino ME, Panizzon MS, Vuoksimaa E, Toomey R (2019) Influence of young adult cognitive ability and additional education on later-life cognition. Proc Natl Acad Sci 116:2021–2026 30670647 10.1073/pnas.1811537116 PMC6369818

[38] Du C, Miyazaki Y, Dong X, Li M (2023) Education, social engagement, and cognitive function: a cross-lagged panel analysis. J Gerontol: Series B 78:1756–1764 10.1093/geronb/gbad088 PMC10561888 37294899

[39] Chen H, Jiang Z, Hu J, Yang X, Gui S, Li Q, Wang J, Yang J (2023) A bidirectional relationship between cognitive reserve and cognition among older adults in a rural Chinese community: a cross-lagged design. Front Psychol 14:1297699 38192390 10.3389/fpsyg.2023.1297699 PMC10773703

[40] Kivipelto M, Mangialasche F, Ngandu T (2018) Lifestyle interventions to prevent cognitive impairment, dementia and Alzheimer disease. Nat Rev Neurol 14:653–666 30291317 10.1038/s41582-018-0070-3

[41] Gavelin HM, Domellöf ME, Leung I, Neely AS, Launder NH, Nategh L, Finke C, Lampit A (2022) Computerized cognitive training in parkinson’s disease: a systematic review and meta-analysis. Ageing Res Rev 80:101671 35714854 10.1016/j.arr.2022.101671

[42] Hindle JV, Martyr A, Clare L (2014) Cognitive reserve in Parkinson’s disease: a systematic review and meta-analysis. Parkinsonism Relat Disord 20:1–7 24034887 10.1016/j.parkreldis.2013.08.010

[43] Liepelt-Scarfone I, Ophey A, Kalbe E (2022) Cognition in prodromal Parkinson’s disease. Cognition in Parkinson’s Disease 269:93 10.1016/bs.pbr.2022.01.003 35248208

[44] Fengler S, Liepelt-Scarfone I, Brockmann K, Schäffer E, Berg D, Kalbe E (2017) Cognitive changes in prodromal Parkinson’s Disease: a review. Mov Disord 32:1655–1666 28980730 10.1002/mds.27135

[45] Jeong SH, Chung SJ, Yoo HS, Jung JH, Baik K, Lee YH, Lee PH, Sohn YH (2022) Premorbid educational attainment and long-term motor prognosis in Parkinson’s Disease. J Parkinsons Dis 12:129–136 34542032 10.3233/JPD-212791

[46] Jefferson AL, Gibbons LE, Rentz DM, Carvalho JO, Manly J, Bennett DA, Jones RN (2011) A life course model of cognitive activities, socioeconomic status, education, reading ability, and cognition. J Am Geriatr Soc 59:1403–1411 21797830 10.1111/j.1532-5415.2011.03499.x PMC3222272

[47] Liu G, Locascio JJ, Corvol J-C, Boot B, Liao Z, Page K, Franco D, Burke K, Jansen IE, Trisini-Lipsanopoulos A (2017) Prediction of cognition in Parkinson’s disease with a clinical–genetic score: a longitudinal analysis of nine cohorts. Lancet Neurol 16:620–629 28629879 10.1016/S1474-4422(17)30122-9 PMC5761650

[48] Chung SJ, Yoo HS, Lee YH, Lee PH, Sohn YH (2019) Heterogeneous patterns of striatal dopamine loss in patients with young-versus old-onset Parkinson’s disease: impact on clinical features. J Mov Disord 12:113 31158944 10.14802/jmd.18064 PMC6547040

[49] Bohnen NI, Yarnall AJ, Weil RS, Moro E, Moehle MS, Borghammer P, Bedard M-A, Albin RL (2022) Cholinergic system changes in Parkinson’s disease: emerging therapeutic approaches. Lancet Neurol 21:381–392 35131038 10.1016/S1474-4422(21)00377-X PMC8985079

[50] Ehrt U, Broich K, Petter J, Ballard C, Aarsland D (2009) Use of drugs with anticholinergic effect and impact on cognition in Parkinson’s disease: a cohort study. J Neurol, Neurosurg Psychiatry 81:160–165 19770163 10.1136/jnnp.2009.186239

