# Supplementary material for: Mid- and late-life lifestyle activities as main drivers of general and domain-specific cognitive reserve in individuals with Parkinson’s disease: cross-sectional and longitudinal evidence from the LANDSCAPE study
Source: J Neurol. 2024 Jul 1;271(8):5411–24. doi: 10.1007/s00415-024-12484-0 (PMC11319368; doi:10.1007/s00415-024-12484-0)
Supplement: Supplementary file 1 — Supplementary file1 (PDF 930 KB) [file 415_2024_12484_MOESM1_ESM.pdf]

Mid- and late-life lifestyle activities as main drivers of general and domain-specific cognitive reserve in individuals with Parkinson's disease: Cross-sectional and longitudinal evidence from the LANDSCAPE study

Supplementary Material

Table S1. Cognitive Tests used in the DEMPRAK/LANDSCAPE Cohort and Their Relevance for the Diagnosis of Parkinson's Disease Mild Cognitive Impairment

Cognitive Domain	Test	Relevant for Level-II Diagnosis	Test battery	Reference
Executive Functions	Semantic Word Fluency	X	CERAD+	Schmid, et al. [1],Morris, et al. [2],Aebi [3]
	Phonemic Word Fluency	X	CERAD+	Schmid, et al. [1],Morris, et al. [2],Aebi [3]
	Trail Making Test B/A		CERAD+	Schmid, et al. [1],Morris, et al. [2],Aebi [3]
	MCST Categories	X		Nelson [4]
	MCST Non-perseverative errors	X		Nelson [4]
	MCST Perseverative errors	X		Nelson [4]
	Digit Span Backward		WMS-R	Härting, et al. [5]
Memory	Word List Learning	X	CERAD+	Schmid, et al. [1],Morris, et al. [2],Aebi [3]
	Word List Recall	X	CERAD+	Schmid, et al. [1],Morris, et al. [2],Aebi [3]
	Figure Recall		CERAD+	Schmid, et al. [1],Morris, et al. [2],Aebi [3]
	Digit Span Forward		WMS-R	Härting, et al. [5]
Attention	Stroop Color Naming	X	-	Bäumler [6]
	Stroop Word Reading	X	-	Bäumler [6]
	Stroop Interference	X	-	Bäumler [6]
	Brief Test of Attention	X	-	Schretlen [7]
Visuospatial	Figure Copy	X	CERAD+	Schmid, et al. [1],Morris, et al. [2],Aebi [3]
	Spatial Rotation: LPS 50+ Subtest 7	X	LPS 50+	Sturm, et al. [8]
	Spatial Imagination: LPS 50 + Subtest 9	X	LPS 50+	Sturm, et al. [8]
Language	Boston Naming Test	X	CERAD+	Schmid, et al. [1],Morris, et al. [2],Aebi [3]

Notes. CERAD+, Consortium to Establish a Registry for Alzheimer's Disease-Plus; LPS, Leistungsprüfungssystem for 50+ aged; MCST, Modified Card Sorting Test; WMS-R, Wechsler Memory Scale-Revised.

Details on the construction of life-stage specific subscores of the Lifetime of Experiences Questionnaire

By equally weighting specific and non-specific scores from each of the three life-stages, three subscores are constructed: young adulthood, mid-life, and late-life. Each subscore combines both the specific and non-specific scores, with each subscore contributing 33.3% to the overall LEQ total score.

The scoring for the present analyses was based on the recently revised German LEQ version (LEQ-D) of Roeske, et al. [9]. To ensure equal weighting of the specific and non-specific scores of each life-stage, normalization factors were applied to adjust for the different value ranges of the life-stage specific LEQ scores. Unlike Valenzuela and Sachdev [10], where these factors are based on the group mean, the normalization factors in the present study were based on the range of item values for each life stage, resulting in sample-independent values. The normalization factors were determined by calculating the quotient of the maximum unspecific score and the maximum specific score. The normalization factor for young adulthood was 1.03 (35/34), for mid-life 0.71 (35/49), and for late-life 0.83 (35/42). The specific scores of each life-stage were then corrected with their corresponding normalization factor before being added to the unspecific score of each life-stage, resulting in the equal weighting of the specific and non-specific score for each life-stage.

Composition of the Lifetime of Experiences Questionnaire (Sub-)Scores

The contribution of complex lifestyle activities in young adulthood, mid-life and late-life either specific or non-specific for the corresponding life-stage to the LEQ total score was analyzed in a 3x2 ANOVA with life-stage (young adulthood vs. mid-life vs. late-life) and specificity (specific vs. non-specific) as within-subjects factors. Post-hoc tests were Bonferroni-adjusted.

There was a significant main effect of life-stage with a medium effect size [$F(2,336)=19.926$, $p<.001$, $\eta_p^2=0.106$], with all pairwise comparisons being significant. LEQ subscores were highest in mid-life (39.73±12.63), followed by young adulthood (36.20±8.75), and then late-life (33.735±8.53). There was a significant main effect of specificity with a small effect size [$F(1,168)=6.459$, $p=.012$, $\eta_p^2=0.037$], overall indicating a higher non-specific contribution (55.41±12.45) compared to a life-stage specific contribution (52.62±15.87).

The interaction of life-stage and specificity was significant with a large effect size [$F(2,336)=35.349$, $p<.001$, $\eta_p^2=0.174$]. Pairwise comparisons reveal no significant difference between the non-specific scores of young adulthood and mid-life and between young adulthood and late-life. For young adulthood, there was no significant difference between the normalized specific score and the non-specific score, indicating an equal contribution of life-stage specific and non-specific activities to total CR. For both mid-life and late-life there were significant differences between the normalized specific score and the non-specific score, however, in opposite directions: For mid-life, the specific score (20.02±10.03) was significantly higher than the non-specific score (18.05±4.08), indicating a stronger contribution of life-stage specific activities (i.e., occupational history) compared to unspecific activities. For late-life, the non-specific score (19.15±6.25) was significantly higher than the specific score (14.58±3.66), indicating a stronger influence of non-life-stage-specific activities (e.g., travel, visiting family, playing musical instruments, doing arts, physical activities, reading, and speaking a foreign language) compared to life-stage specific social and intellectual activities.

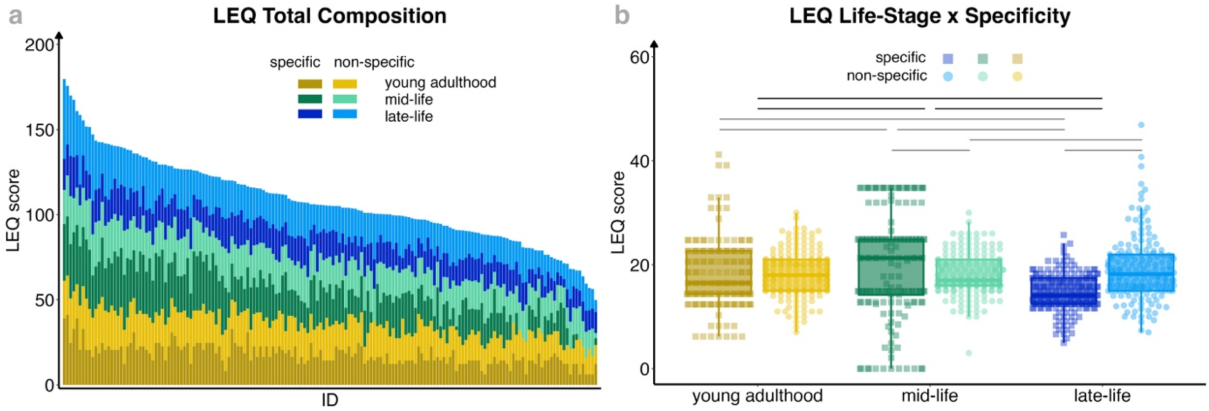

Fig. S1 Lifetime of Experience Questionnaire scores by contributing life-stage and specificity

a. Composition of the baseline scores of the Lifetime of Experiences Questionnaire (LEQ) by life-stage (young adulthood, mid-life, late-life) and specificity (specific vs. non-specific) regarding the lifestyle activities for the corresponding life-stage. b. Comparison of the LEQ scores by life-stage and specificity. Dots and squares represent individual LEQ subscores, the group-wise boxplots visualize the within-group median, the hinges represent the corresponding first and the third quartile, and the whiskers are 1.5 * the inter-quartile range. Lines indicate significant ($p < .05$) Bonferroni-adjusted pairwise comparisons for the main effect of life-stage (black) and the interaction of life-stage and specificity (grey).

Table S2. Sociodemographic, clinical, and cognitive characteristics of the DEMPARK/LANDSCAPE sample: completed study vs. lost to attrition.

	**ALL** (*n* = 169)	**Last possible follow-up completed** (*n* = 64)	**Dropout at last possible follow-up** (*n* = 105)	*p*-value
**Age** in years	70.67 (6.5) [50-85]	69.28 (6.73) [50-82]	71.52 (6.23) [50-85]	.029
**Sex**, n (%)	M: 120 (71.0) F: 49 (29.0)	M: 45 (70.3) F: 19 (29.7)	M: 75 (71.4) F: 30 (28.6)	.999
**Education** in years	14.24 (2.93) [8-20]	14.12 (2.87) [8-20]	14.31 (2.98) [8-20]	.685
**PANDA**	24.06 (4.55) [8-30]	23.48 (5.03) [8-30]	24.42 (4.2) [11-30]	.198
**MMSE**	28.64 (1.39) [22-30]	28.52 (1.44) [24-30]	28.71 (1.36) [22-30]	.405
**Disease duration** in months	98.63 (48.27) [25-308]	93.69 (42.8) [25-232]	101.67 (51.31) [26-308]	.299
**Age at PD diagnosis** in years	62.38 (7.47) [42-75]	61.52 (7.59) [42-73]	62.91 (7.39) [43-75]	.240
**UPDRS-III**	24.78 (11.91) [3-60]	23.57 (9.9) [7-55]	25.51 (12.98) [3-60]	.318
**LEDD**	655.58 (384.09) [52-2120]	642.75 (395.89) [52-1837.5]	663.4 (378.43) [54-2120]	.736
**Depressive symptoms (GDS)**	3.01 (2.88) [0-15]	3.14 (2.64) [0-11]	2.93 (3.02) [0-15]	.649
**CERAD-Plus** Total Score	0.03 (0.63) [-2.41-1.44]	0.1 (0.57) [-2.01-1.37]	-0.01 (0.67) [-2.41-1.44]	.252
**Executive** composite	-0.63 (0.79) [-2.56-1.13]	-0.55 (0.74) [-2.31-0.94]	-0.68 (0.82) [-2.56-1.13]	.321
**Memory** composite	-0.4 (0.82) [-3.09-1.27]	-0.28 (0.7) [-2.31-1.2]	-0.48 (0.89) [-3.09-1.27]	.135
**Attention** composite	-0.55 (1.16) [-4-1.33]	-0.56 (1.07) [-3.18-1.03]	-0.54 (1.22) [-4-1.33]	.904
**Visuospatial** composite	-0.4 (0.77) [-3.5-1.14]	-0.33 (0.65) [-3.1-1.12]	-0.45 (0.83) [-3.5-1.14]	.366
**Language** composite	0.29 (0.88) [-2.7-1.78]	0.32 (0.9) [-2.7-1.78]	0.27 (0.87) [-2.33-1.54]	.753

Notes. Data are mean and standard deviation unless indicated otherwise. *p*-values of independent sample *t*-tests or χ^2-tests between individuals with PD-N and PD-MCI & PDD are presented. CERAD-Plus Total Score, Consortium to establish a Registry of Alzheimer´s Disease Plus Total Score; F, female; GDS, Geriatric Depression Scale; LEDD, Levodopa Equivalent Daily Dose; LEQ, Lifetime of Experiences Questionnaire; M: male; MMSE, Mini-Mental State Examination; PANDA, Parkinson Neuropsychometric Dementia Assessment; PD, Parkinson's disease; UPDRS-III, Unified Parkinson´s Disease Rating Scale Part 3.

Life-stage specific vs. non-specific associations with cognitive performance over time

To get a more detailed understanding of life-stage specific vs. non-specific associations with cognitive performance over time, we built a bivariate correlation matrix of all LEQ subscores (life-stage x specificity) (life-stage x specificity) with cross-sectional cognitive performance at LEQ baseline as well as the difference scores of cognitive performance per year follow-up compared to the LEQ baseline (Supplementary Figure S2). The strongest and most consistent correlations for cross-sectional cognitive performance were found for non-specific mid-life lifestyle activities and specific late-life lifestyle activities. The bivariate association with cognitive change over time is weaker, with a tendency for specific young adulthood proxies of CR (which is mainly determined by education in the LEQ) showing stronger negative associations with cognitive performance.

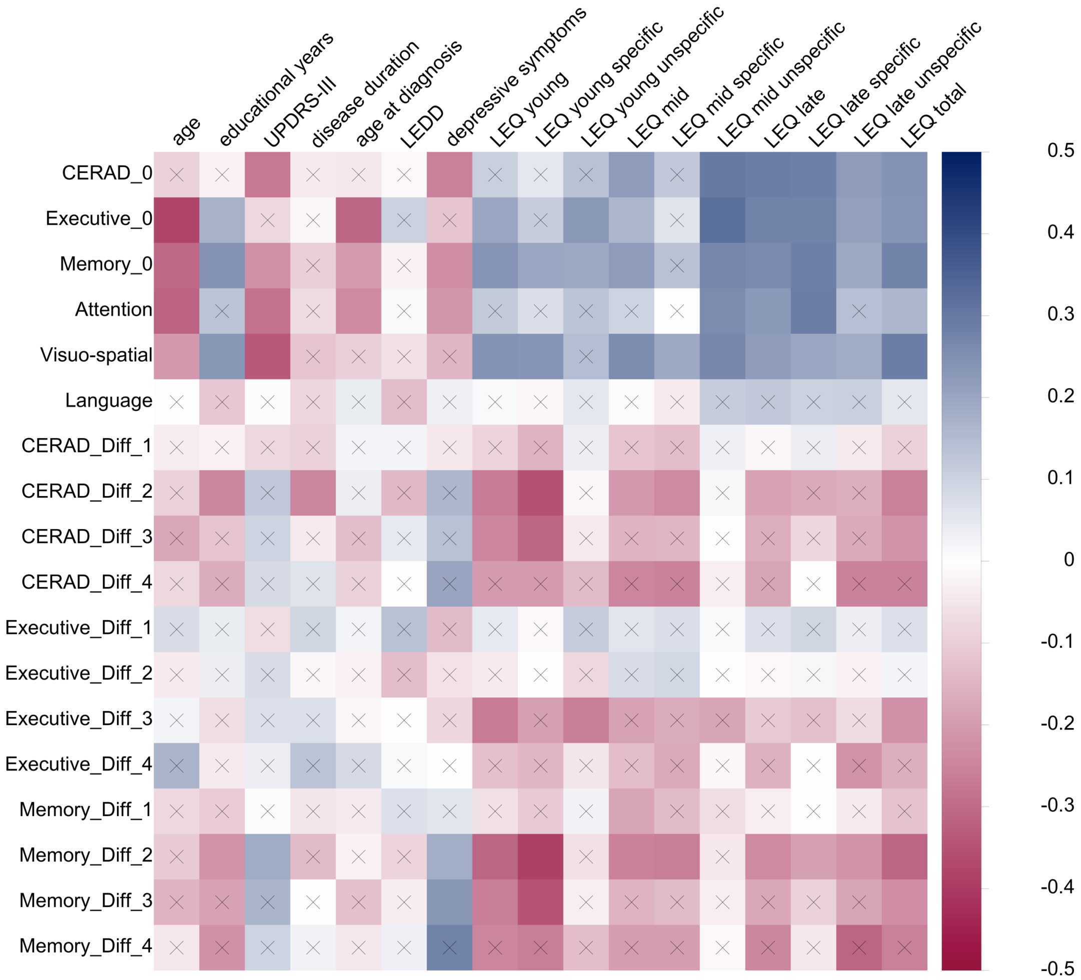

Fig. S2 Bivariate Pearson correlation matrix of demographic and clinical variables and the Lifetime of Experiences Questionnaire subscores with cognitive performance at baseline and longitudinal difference scores

The color spectrum represents the Peason correlation coefficient from min. -0.5 (dark red) to max. 0.5 (dark blue). Crosses indicate non-significant (p≥.05) correlations. LEDD, Levodopa Equivalent Daily Dose; LEQ, Lifetime of Experiences Questionnaire.

Table S3. Linear mixed effect models evaluating the influence of cognitive reserve on cognitive performance over time (max. 3-year follow-up).

	**CERAD Total Score**			**Executive Functions**			**Memory**			**Attention**			**Visuospatial Functioning**		
**Fixed effects**	*Estimates*	β	*p*	*Estimates*	β	*p*	*Estimates*	β	*p*	*Estimates*	β	*p*	*Estimates*	β	*p*
Intercept	0.521 (0.255 – 0.786)		**<.001**	-0.454 (-0.727 – -0.181)		**.003**	0.112 (-0.210 – 0.433)		.495	0.237 (-0.260 – 0.735)		.349	0.230 (-0.098 – 0.558)		.170
Time	-0.087 (-0.124 – -0.050)	-0.154	**<.001**	-0.135 (-0.182 – -0.089)	-0.176	**<.001**	-0.057 (-0.105 – -0.009)	-0.076	**.020**	-0.242 (-0.312 – -0.172)	-0.206	**<.001**	-0.091 (-0.131 – -0.051)	-0.123	**<.001**
LEQ Total	0.004 (0.000 – 0.007)	0.134	**.047**	0.010 (0.006 – 0.013)	0.282	**<.001**	0.009 (0.004 – 0.013)	0.256	**<.001**	0.008 (0.001 – 0.015)	0.145	**.033**	0.008 (0.003 – 0.012)	0.230	**.001**
Time*LEQ Total	-0.001 (-0.003 – -0.000)	-0.090	.060	-0.002 (-0.004 – 0.000)	-0.086	.064	-0.002 (-0.004 – -0.000)	-0.107	**.029**	0.001 (-0.002 – 0.003)	0.019	.666	-0.001 (-0.002 – 0.001)	-0.035	.397
Age	-0.006 (-0.019 – 0.008)	-0.061	.388	-0.041 (-0.054 – -0.027)	-0.319	**<.001**	-0.040 (-0.056 – -0.024)	-0.318	**<.001**	-0.055 (-0.080 – -0.030)	-0.282	**<.001**	-0.029 (-0.046 – -0.013)	-0.236	**<.001**
Sex: Female	0.091 (-0.111 – 0.292)	0.070	.333	0.228 (0.023 – 0.433)	0.123	**.029**	0.212 (-0.028 – 0.451)	0.116	.083	0.323 (-0.059 – 0.705)	0.113	.097	-0.203 (-0.449 – 0.044)	-0.111	.107
Diagnosis: PD-MCI	-0.518 (-0.698 – -0.338)	-0.405	**<.001**	-0.803 (-0.987 – -0.619)	-0.475	**<.001**	-0.486 (-0.701 – -0.272)	-0.294	**<.001**	-0.624 (-0.964 – -0.284)	-0.239	**<.001**	-0.395 (-0.616 – -0.174)	-0.240	**<.001**
Disease Duration	-0.000 (-0.002 – 0.002)	0.004	.953	0.001 (-0.001 – 0.002)	0.031	.529	-0.001 (-0.003 – 0.001)	-0.067	.261	0.000 (-0.003 – 0.004)	0.016	.785	-0.001 (-0.003 – 0.001)	-0.042	.507
UPDRS-III	-0.007 (-0.015 – 0.001)	-0.129	.054	0.005 (-0.003 – 0.013)	0.067	.197	-0.006 (-0.015 – 0.003)	-0.076	.219	-0.018 (-0.032 – -0.003)	-0.150	**.015**	-0.014 (-0.023 – -0.005)	-0.191	**.003**
Depressive Symptoms	-0.027 (-0.059 – 0.006)	-0.116	.106	-0.011 (-0.044 – 0.022)	-0.036	.523	-0.029 (-0.068 – 0.010)	-0.098	.140	-0.056 (-0.118 – 0.005)	-0.121	.072	0.004 (-0.035 – 0.044)	0.015	.828
**Random effects**															
σ^2	0.07			0.15			0.14			0.19			0.15		
τ_{00}	0.23_{ID}			0.19_{ID}			0.34_{ID}			0.85_{ID}			0.34_{ID}		
τ_{11}	$0.02_{\text{ID.time.num}}$			$0.02_{\text{ID.time.num}}$			$0.03_{\text{ID.time.num}}$			$0.09_{\text{ID.time.num}}$			$0.01_{\text{ID.time.num}}$		
ρ_{01}	0.04_{ID}			0.31_{ID}			-0.16_{ID}			0.17_{ID}			0.10_{ID}		
ICC	0.81			0.68			0.73			0.86			0.72		
N	157_{ID}			157_{ID}			157_{ID}			157_{ID}			156_{ID}		
Observations	440			461			462			447			467		
Marginal R^2 / Conditional R^2	0.244 / 0.854			0.408 / 0.808			0.280 / 0.804			0.249 / 0.897			0.248 / 0.791		

Notes. Data are unstandardized coefficients and (95% confidence intervals). Furthermore, standardized β coefficients and corresponding *p*-values are reported. LEQ subscores and age were mean-centered prior to model estimation. ICC, intraclass correlation coefficient, LEQ, Lifetime of Experiences Questionnaire; UPDRS-III, Unified Parkinson's Disease Rating Scale Part 3; σ^2, variance of residual errors; τ_{00}, variance of the random intercepts; τ_{11}, variance of the random slopes; ρ_{01}, covariance between random intercepts and random slopes.

References

1. Schmid NS, Ehrensperger MM, Berres M, Beck IR, Monsch AU (2014) The Extension of the German CERAD Neuropsychological Assessment Battery with Tests Assessing Subcortical, Executive and Frontal Functions Improves Accuracy in Dementia Diagnosis. Dement Geriatr Cogn Dis Extra 4:322-334
2. Morris J, Heyman A, Mohs R, Hughes J, van Belle G, Fillenbaum G, Mellits E, Clark C (1989) The Consortium to Establish a Registry for Alzheimer's Disease (CERAD). Part I. Clinical and neuropsychological assesment of Alzheimer's Disease. Neurology 39:1159-1159
3. Aebi C (2002) Validierung der neuropsychologischen Testbatterie CERAD-NP: eine Multi-Center Studie. In:University of Basel, Basel
4. Nelson HE (1976) A modified card sorting test sensitive to frontal lobe defects. Cortex 12:313-324
5. Härting C, Markowitsch H-J, Neufeld H, Calabrese P, Deisinger K, Kessler J (2000) WMS-R, Wechsler memory scale, revised version. In:
6. Bäumler G (1985) Farb-Wort-Interferenztest (FWIT) nach J.R. Stroop. In:Hogrefe, Göttingen
7. Schretlen D (ed) (1997) Brief Test of Attention (BTA). Florida
8. Sturm W, Willmes K, Horn W (1993) Leistungsprüfungssystem für 50-90jährige (LPS50+).
9. Roeske S, Wolfsgruber S, Kleineidam L, Zulka L, Buerger K, Ewers M, Laske C, Nestor P, Peters O, Priller J (2018) P3-591: A GERMAN VERSION OF THE LIFETIME OF EXPERIENCES QUESTIONNAIRE (LEQ) TO MEASURE COGNITIVE RESERVE: VALIDATION RESULTS FROM THE DELCODE STUDY. Alzheimer's & Dementia 14:P1352-P1353
10. Valenzuela MJ, Sachdev P (2007) Assessment of complex mental activity across the lifespan: development of the Lifetime of Experiences Questionnaire (LEQ). Psychological medicine 37:1015-1025
